# Oleuropein Promotes Neural Plasticity and Neuroprotection via PPARα-Dependent and Independent Pathways

**DOI:** 10.3390/biomedicines11082250

**Published:** 2023-08-11

**Authors:** Foteini Malliou, Christina E. Andriopoulou, Aristeidis Kofinas, Allena Katsogridaki, George Leondaritis, Frank J. Gonzalez, Theologos M. Michaelidis, Marousa Darsinou, Leandros A. Skaltsounis, Maria Konstandi

**Affiliations:** 1 Department of Pharmacology, Faculty of Medicine, University of Ioannina, 45110 Ioannina, Greece; f.malliou@ifet.gr (F.M.); xristina_an22@hotmail.com (C.E.A.); ar.kofinas@gmail.com (A.K.); alexkatsogridaki@gmail.com (A.K.); gleondar@uoi.gr (G.L.); 2 Institute of Biosciences (I.BS.), University Research Center of Ioannina (U.R.C.I.), 45110 Ioannina, Greece; 3 Laboratory of Metabolism, National Cancer Institute, National Institutes of Health, Bethesda, MD 20814, USA; gonzalef@mail.nih.gov; 4 Department of Biological Applications & Technology, School of Health Sciences, University of Ioannina, 45110 Ioannina, Greece; tmichael@uoi.gr (T.M.M.); m.darsinou@ucl.ac.uk (M.D.); 5 Biomedical Research Institute, Foundation for Research and Technology-Hellas, 45110 Ioannina, Greece; 6 Department of Pharmacognosy, Faculty of Pharmacy, National and Kapodestrian University of Athens, 11527 Athens, Greece; skaltsounis@pharm.uoa.gr

**Keywords:** oleuropein, neural plasticity, BDNF, PPARα, neurotrophin

## Abstract

Oleuropein (OLE), a main constituent of olives, displays a pleiotropic beneficial dynamic in health and disease; the effects are based mainly on its antioxidant and hypolipidemic properties, and its capacity to protect the myocardium during ischemia. Furthermore, OLE activates the peroxisome proliferator-activated receptor (PPARα) in neurons and astrocytes, providing neuroprotection against noxious biological reactions that are induced following cerebral ischemia. The current study investigated the effect of OLE in the regulation of various neural plasticity indices, emphasizing the role of PPARα. For this purpose, 129/Sv wild-type (WT) and *Pparα*-null mice were treated with OLE for three weeks. The findings revealed that chronic treatment with OLE up-regulated the brain-derived neurotrophic factor (BDNF) and its receptor TrkB in the prefrontal cortex (PFC) of mice via activation of the ERK1/2, AKT and PKA/CREB signaling pathways. No similar effects were observed in the hippocampus. The OLE-induced effects on BDNF and TrkB appear to be mediated by PPARα, because no similar alterations were observed in the PFC of *Pparα*-null mice. Notably, OLE did not affect the neurotrophic factors NT3 and NT4/5 in both brain tissues. However, fenofibrate, a selective PPARα agonist, up-regulated BDNF and NT3 in the PFC of mice, whereas the drug induced NT4/5 in both brain sites tested. Interestingly, OLE provided neuroprotection in differentiated human SH-SY5Y cells against β-amyloid and H_2_O_2_ toxicity independently from PPARα activation. In conclusion, OLE and similar drugs, acting either as PPARα agonists or via PPARα independent mechanisms, could improve synaptic function/plasticity mainly in the PFC and to a lesser extent in the hippocampus, thus beneficially affecting cognitive functions.

## 1. Introduction

Over the last twenty years, intensive research indicated that the revolution in adult brain functionality largely depended on neural plasticity, a property describing the ability of the brain to adapt to various intrinsic and extrinsic stimuli by reorganizing its structure, function and connections [1,2,3].

Accumulating evidence suggests that neurotrophins (NTs) along with their cognate tyrosine kinase receptors (Trks) hold key roles in neural plasticity, thus determining the development and function of the nervous system [4,5]. They belong to a family of neurotrophic factors that are secreted by presynaptic and postsynaptic neurons, microglia and glial cells, astrocytes and oligodendrocytes or other types of cells including muscle cells, and display paracrine and autocrine actions on these cells [6,7,8,9,10,11,12,13,14,15]. In particular, NTs determine the development of neuronal networks by regulating the growth of neuronal processes, the development of synapses and synaptic plasticity, as well as neuronal survival, differentiation and myelination. They decisively affect cognitive functions, such as learning and memory, brain development and homeostasis, sensorial training and recovery from brain injury [1].

NTs exert their effects on the central and sympathetic nervous system via activation of their specific tyrosine kinase (TrkA, TrkB and TrkC) receptors belonging to the family of tropomyosin-related kinase receptors [16,17]. Brain-derived neurotrophic factor (BDNF) is the most abundant neurotrophin in the adult brain, and is one of the major regulators of neurotransmission and neural plasticity [13,18,19]. It is an essential regulator of cellular signaling that underlies cognition, and in particular, synaptic efficacy, which is a determinant parameter in learning and memory [20]. Deficits in BDNF signaling are associated with the pathogenesis of various neurological and psychiatric disorders, including Alzheimer’s disease (AD) and depression [20,21,22], whereas BDNF administration attenuates amyloid-β peptide-induced memory deficits [23].

Neurotrophin 3 (NT3) is structurally related to BDNF and is linked to neurogenesis mainly in the hippocampus, as it promotes hippocampal cell growth, differentiation and survival. NT3 has also neuroprotective effects on sympathetic and sensory neurons [24,25]. Neurotrophin 4/5 (NT4/5) also plays a significant role in neurogenesis [26], and affects neuritic morphology and synapse formation [27]. It is worth noting that BDNF, NT3 and NT4/5 levels are decreased in the hippocampus of AD patients [28].

Normal aging and neurodegenerative disorders are usually associated with cognitive deficits, and researchers focus on finding compounds that could prevent, delay or restore this cognitive deterioration. Polyphenols, such as resveratrol, isolated from medicinal plants, are intensively studied due to their beneficial effects on memory and neuroprotection [29]. Other polyphenolic compounds, including curcumin [30,31], fisetin [31] and epicatechin [32], were found to improve synaptic plasticity.

Oleuropein (OLE) and its hydrolysis product, hydroxytyrosol, are the main constituents of the leaves and unprocessed olive drupes of *Olea europaea*. Preclinical studies reported that these compounds display pleiotropic health benefits, mainly associated with their cardioprotective [33,34,35,36,37,38,39,40], anti-inflammatory [41], anti-diabetic [42] and anti-oxidant properties [43]. Current studies employing murine models of AD indicated that OLE also displays neuroprotective properties [44,45]. In particular, OLE ameliorated cognitive impairment and improved synaptic function in TgCRND8 mice, a well-known model of Alzheimer’s disease expressing a double mutant form of human amyloid-β precursor protein via inhibition of β-amyloid peptide aggregation, which is associated with neural toxicity [46]. Furthermore, OLE prevented the colchicine-induced cognitive dysfunction in rats [47]. It is also of note that long-term treatment of old mice with phenol-rich extra-virgin olive oil improved their memory and learning ability [48]. Olive oil also improved the performance of senescence accelerated mouse-prone (SAMP8) mice, a naturally occurring model of accelerated aging, in the T-maze test [49].

A previous study reported that fenofibrate (FEN), a PPARα agonist, markedly activated the hippocampal peroxisome proliferator-activated receptor gamma coactivator-1 alpha (PGC-1α)/irisin/BDNF pathway, and induced synaptic plasticity in rats following a high-fat, high-fructose diet [50]. Therefore, the present study investigated the effect of OLE, a PPARα agonist [39], on neural plasticity, emphasizing the role of this nuclear transcription factor in the OLE-mediated regulation of the neurotrophins, BDNF, NT3 and NT4/5. For this purpose, 129/Sv wild-type (WT) and *Pparα*-null mice were treated with either OLE or FEN, a selective PPARα agonist. The data indicated that OLE up-regulated BDNF and its receptor TrkB in the PFC of mice, while it had no effect in their hippocampus, though it increased the synthesis of NT4/5. FEN also triggered a strong induction of BDNF, NT3 and TrkB in the PFC of mice, and increased the synthesis of NT4/5 in their hippocampus and PFC. The OLE- and FEN-induced effects on these important neural plasticity factors were PPARα-dependent, because they did not occur in the PPARα-deficient mice.

## 2. Materials and Methods

### 2.1. Animals

Adult male 129/Sv WT and *Ppara*-null mice [51,52] were used in this study. The WT and *Pparα*-null mice received *ad libitum* the standard rodent chow diet (diet 1324 TPF, Altromin Spezialfutter GmbH & Co., KG, Lage, Germany). All of the animals were housed up to five per cage under a standard 12-h light, 12-h dark cycle, and had continuous access to drinking water. The mice were monitored on a daily basis for outward signs of distress or adverse health effects. All procedures involving the animals were reviewed and approved by the ethics committee of the Medical School of the University of Ioannina. They conformed to the International European Ethical Standards (86/609-EEC) for the care and use of laboratory animals.

### 2.2. Drugs and Treatment

OLE (100 mg/kg) was administered daily in the food pellets for three consecutive weeks. The dosing regime of OLE was designed using findings from previous dose–response experiments [40]. At the end of the experiment, the mice were killed by CO_2_ asphyxiation, and trunk blood was collected in BD microtainer serum separator tubes (Becton, Dickinson and Company, Franklin Lakes, NJ, USA) for biochemical analyses. The hippocampus and prefrontal cortex were dissected from the brain for total RNA and total cellular protein extraction. All of the brain tissues and serum samples were kept at −80 °C until assayed.

### 2.3. In Vivo Experiments

Adult male (129/Sv) WT and *Ppara*-null mice were randomly assigned into groups of 8–10 mice. OLE (100 mg/kg) was administered daily in the food pellets for three consecutive weeks in both WT and *Ppara*-deficient mice. The controls received the normal rodent diet.

The dose of OLE was based on our previous findings [40,53] and on the literature [54]. OLE was administered in the food pellets, because it has been shown that even under normal iso-osmotic luminal conditions, OLE is poorly absorbed. Its absorption can be significantly improved by solvent flux through paracellular junctions, made possible by hypotonic conditions in the intestinal lumen [55]. The presence of glucose or amino acids in the intestinal lumen that follows a meal stimulates water flux via the opening of paracellular junctions. It is possible that this mechanism has a similar effect on OLE absorption as a hypotonic solution [56]. Although the pharmacokinetic profile of OLE has not been determined in mice, Boccio and colleagues indicated that a single oral dose of OLE (100 mg/kg) is absorbed in rats, reaching 200 ng/mL in tmax of 2 h [57]. The experiment was terminated when the mice of all groups were killed with CO_2_ asphyxiation.

### 2.4. Quantitative Real-Time PCR

The total RNA from the hippocampus and PFC was isolated using Trizol reagent (Invitrogen, Carlsbad, CA, USA), following the manufacturer’s protocol. The concentration of total RNA in each sample was determined spectrophotometrically. Quantitative real-time PCR (qPCR) was performed with cDNA, which was generated from 1 μg of total RNA using a SuperScript II reverse transcriptase kit (Invitrogen). The sequences of the forward and reverse gene-specific primers that were used in this study are shown in Table 1. The SYBR Green PCR Master Mix (Applied Biosystems, Foster City, CA, USA) was used for the real-time reactions, which were performed employing a C1000 Touch thermal cycler with a real-time detection system (Bio-Rad Laboratories, Hercules, CA, USA). The relative mRNA expression was normalized to β-actin levels (QuantiTect primer assay; QIAGEN, Valencia, CA, USA), and the values were quantified using the comparative threshold cycle method.

### 2.5. Western Blot Analysis

Immunoblot analysis of BDNF, TrkB, phospho-ERK, phospho-CREB and phospho-AKT protein levels was performed using total cellular extracts from the hippocampus, PFC and differentiated into neurons of human SH-SY5Y neuroblastoma cells. The total cellular proteins were extracted using the RIPA buffer supplemented with protease inhibitors, phenylmethylsulfonyl fluoride (1 mΜ), β-glycerophosphate (5 mΜ), NaF (5 mM), Na_2_MoO_4_ (2 mM) and NaVO_3_ (1 mM). The BCA protein assay kit (Pierce, IL, USA) was used for the determination of the protein concentration in the samples. The proteins were subjected to SDS-polyacrylamide gel electrophoresis and immunoblotting using the following antibodies: rabbit polyclonal BDNF-specific IgG (Santa Cruz Biotechnology, Dallas, TX, USA), rabbit polyclonal phosphorylated (Ser133) CREB-1-specific IgG (cell signaling, Danvers, MA, USA), rabbit monoclonal TrkB (cell signaling), rabbit monoclonal phospho-ERK (cell signaling) and rabbit monoclonal phospho-AKT (cell signaling). Secondary antibodies conjugated with horseradish peroxidase (Santa Cruz Biotechnology) were used, and the proteins were detected using an enhanced chemiluminescence detection kit (GE Healthcare, Chalfont St. Giles, Buckinghamshire, UK). Immunoblotting with either α-tubulin- or β-actin-specific antibodies (Santa Cruz Biotechnology) and anti-goat IgG horseradish peroxidase-conjugated secondary antibody was used as a loading control.

### 2.6. In Vitro Experiments

#### 2.6.1. Cell Culture

The human neuroblastoma cell line, SH-SY5Y, was obtained from the American Type Culture Collection (ATCC, Manassas, VA, USA). The SH-SY5Y cells were cultured in a mixture of Dulbecco’s modified Eagle’s medium (DMEM GlutaMAX) and Ham’s F12 NutrientMix (1:1), containing glucose (25 mM) and l-glutamine (2 mM). This medium was supplemented with 10% (*v*/*v*) heat-inactivated fetal bovine serum (FBS) and 1% penicillin–streptomycin (P/S). The cells kept at a confluence below 70% (100,000 cells per well) were cultivated in 12-well plates at 37 °C with 5% CO_2_ and saturated humidity.

#### 2.6.2. Cell Differentiation and Viability

Following 24 h of incubation, the human neuroblastoma SH-SY5Y cells were differentiated into cholinergic neurons using retinoic acid (RA, 5 μM), which was added to culture medium that did not contain FBS. The cell incubation lasted for 5 days, and the culture medium supplemented with fresh RA was changed every three days.

Following 5 days of incubation, differentiated human SH-SY5Y cells were treated for 4 h with the culture medium without FBS, containing only either OLE (10 μM) or fenofibrate (FEN, Sigma-Aldrich, Burghausen, Germany, 10 μM), or the highly selective PPARα agonist, 4-Chloro-6-(2,3-xylidino)-2-pyrimidinylthioacetic acid (Wy-14643, Sigma-Aldrich, Germany, 10 μM). Subsequently, either H_2_O_2_ (final concentration 750 μΜ) or 7PA2-CHO supernatant (final concentration 75% *v*/*v*) from a CHO (Chinese hamster ovary) cell line stably expressing a mutant form of the human amyloid precursor protein (APP) [58] was added in the cell culture, in order to induce β-amyloid toxicity. CHO-K1 supernatant at a final concentration of 75% *v/v* was used in the control cell cultures. Following incubation of the differentiated cells for an additional 48 h, MTS reagent [20 μL of MTS (1.90 mg/mL)] was added, and the plates were incubated at 37 °C and 5% CO_2_ for 120 min. The absorbance was measured using a spectrophotometer at 492 nm, and the results were expressed as % cell viability compared to DMSO- or medium-treated cells, which represented 100% of cell viability.

### 2.7. Statistical Analysis

The present data are presented as the mean ± SE. They were analyzed using the one-way analysis of variance (ANOVA) program, followed by multiple comparisons employing the Bonferonni’s and Tuckey’s list honest significant difference methods. The significance level for all of the analyses was set at probability of less than 0.05.

## 3. Results

### 3.1. Assessment of Chronic OLE Treatment in Neural Plasticity Indices in PFC

**Figure 1:**
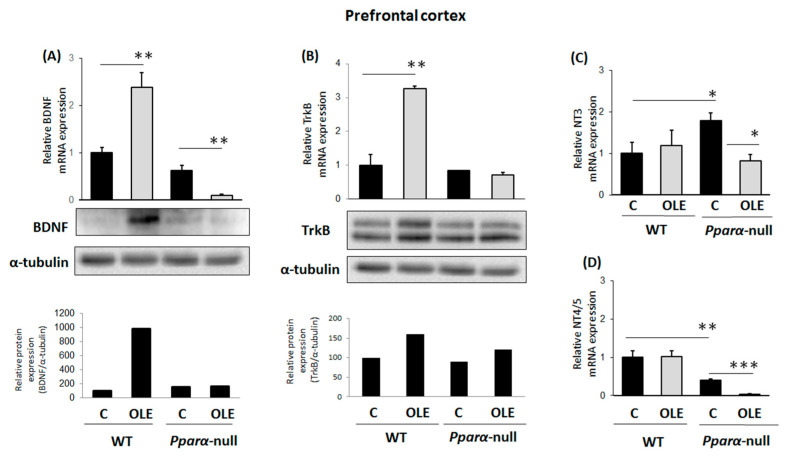
The effect of chronic treatment with oleuropein (OLE) on neural plasticity indices in the prefrontal cortex (PFC) of mice. (**A**) Following treatment with OLE, brain-derived neurotrophic factor (BDNF) mRNA levels were analyzed in 129/Sv wild-type (WT) and *Pparα*-null mice by qPCR and protein levels using Western blot. (**B**) TrkB receptor mRNA and protein levels were also analyzed using qPCR and Western blot analysis, respectively. (**C**) Neurotrophin NT3 and (**D**) NT4/5 mRNA levels were also analyzed with qPCR. Values were normalized to β-actin, and are expressed as mean ± SE (n = 8–10). Comparisons were between controls C and OLE-treated mice. Treatment group differences were calculated using one-way ANOVA, followed by Bonferroni’s test. * *p* < 0.05, ** *p* < 0.01, *** *p* < 0.001.

The treatment of WT mice with OLE, a PPARα agonist [39], for 21 days increased BDNF mRNA and protein expression in their PFCs. This effect was *Pparα*-dependent, because OLE did not increase *BDNF* in *Ppara*-null mice (Figure 1A). Similarly, chronic treatment of WT mice with OLE increased TrkB mRNA and protein expression in their PFCs, an effect apparently involving PPARα, because no change in *TrkB* expression was observed in this brain region of *Pparα*-null mice (Figure 1B). Notably, OLE repressed BDNF mRNA and protein expression in the PFC of *Pparα*-deficient mice (Figure 1A), an effect that underscores the distinct role of PPARα in the effects of OLE on BDNF in this brain area. Chronic treatment of WT mice with OLE had no effect on *NT3* and *NT4/5* mRNA expression in their PFCs (Figure 1C and Figure 1D, respectively). Interestingly, baseline *NT3* mRNA levels were higher in *Pparα*-null than in WT mice, and OLE repressed them (Figure 1C). Constitutive *NT4/5* mRNA expression raged at lower levels in the PFC of *Pparα*-null mice than in WT mice, and OLE further repressed it (Figure 1D).

### 3.2. Assessment of Chronic OLE Treatment on Neural Plasticity Indices in the Hippocampus

**Figure 2:**
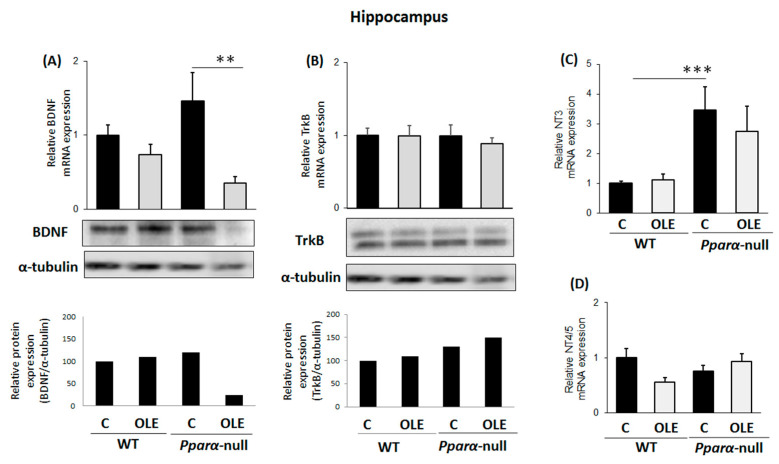
The effect of chronic treatment with oleuropein (OLE) on neural plasticity indices in the hippocampus of mice. (**A**) Following treatment with OLE, brain-derived neurotrophic factor (BDNF) mRNA levels were analyzed in 129/Sv wild-type and *Pparα*-null mice using qPCR, and protein levels using Western blot. (**B**) TrkB receptor mRNA and protein levels were also analyzed using qPCR and Western blot analysis, respectively. (**C**) Neurotrophin NT3 and (**D**) NT4/5 mRNA levels were also analyzed with qPCR. Values were normalized to β-actin, and are expressed as mean ± SE (n = 8–10). Comparisons were between controls C and OLE-treated mice. Treatment group differences were calculated using one-way ANOVA, followed by Bonferroni’s test. ** *p* < 0.01, *** *p* < 0.001.

Chronic treatment of WT mice with OLE did not affect BDNF and TrkB mRNA and protein expression in their hippocampuses (Figure 2A and Figure 2B, respectively). Importantly, however, OLE significantly repressed BDNF mRNA and protein expression in the hippocampus of *Pparα*-deficient mice (Figure 2A), an effect that indicates the preventive role of PPARα in the OLE-mediated down-regulation of BDNF in the hippocampus. In WT mice following chronic OLE treatment, no alteration was observed in *NT3* mRNA expression in their hippocampuses compared to controls (Figure 2C). Notably, constitutive *ΝΤ3* mRNA expression ranged at higher levels in the hippocampus of *Pparα*-null mice than in WT mice (Figure 2C). OLE did not affect *NT4/5* mRNA expression in the hippocampus of WT and *Pparα*-null mice (Figure 2D).

### 3.3. OLE-Induced ERK, AKT and PKA/CREB Activation

**Figure 3:**
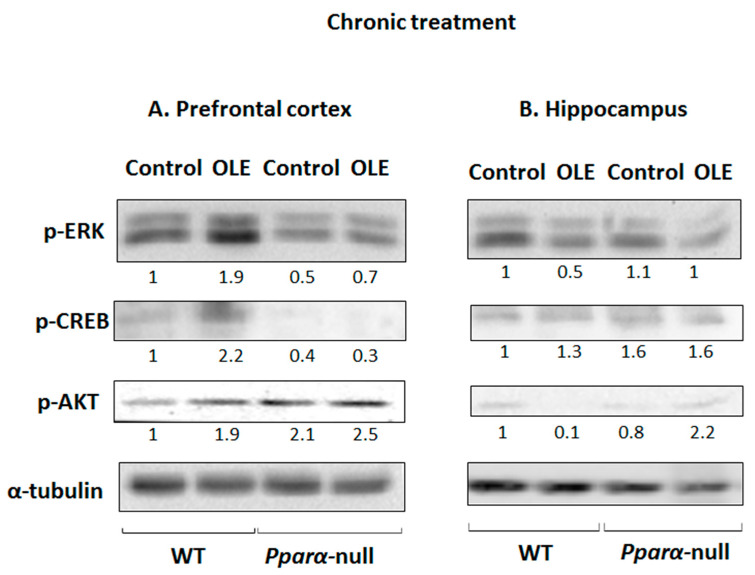
The role of chronic treatment with oleuropein (OLE) in the activation of ERK1/2, PKA/CREB and PI3K/AKT signaling pathways. Phosphorylated ERK1/2, CREB and AKT expression levels were examined in proteins extracted from the prefrontal cortex and hippocampus of mice using Western blot analysis. WT: wild-type mice. The numbers underneath the lanes represent the relative protein expressions that are defined as the ratio between the OLE-treated and control expression, which is set at 1.

Chronic treatment of WT mice with OLE increased the phosphorylation of ERK1/2, CREB and AKT in their PFCs compared to controls (Figure 3A). The role of PPARα in ERK1/2, CREB and AKT activation by OLE appears to be determinant, because the drug did not affect the activation of these signaling pathways in *Pparα*-null mice (Figure 3A). Interestingly, pCREB protein levels were markedly lower in the PFCs of *Pparα*-deficient mice, and OLE did not affect them (Figure 3A). OLE had no similar activating effects on ERK1/2-, AKT- and PKA/CREB-linked signaling pathways in the hippocampus of WT mice (Figure 3B).

### 3.4. Assessment of Subacute OLE Treatment in Neural Plasticity Indices in PFC

**Figure 4:**
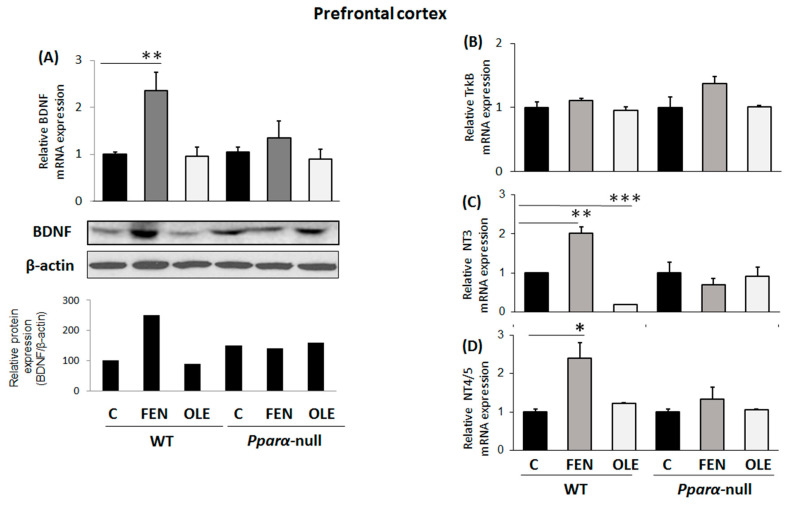
The effect of subacute treatment with oleuropein (OLE) on neural plasticity indices in the prefrontal cortex (PFC) of mice. (**A**) Following treatment with either OLE or fenofibrate (FEN), brain-derived neurotrophic factor (BDNF) mRNA levels were analyzed in 129/Sv wild-type (WT) and *Pparα*-null mice using qPCR, and protein levels using Western blot. (**B**) TrkB receptor mRNA and protein levels were also analyzed using qPCR and Western blot analysis, respectively. (**C**) Neurotrophin NT3 and (**D**) NT4/5 mRNA levels were also analyzed with qPCR. Values were normalized to β-actin and are expressed as mean ± SE (n = 8–10). Comparisons were between controls C and OLE-treated mice. Treatment group differences were calculated using one-way ANOVA, followed by Bonferroni’s test. * *p* < 0.05, ** *p* < 0.01, *** *p* < 0.001.

The treatment of WT mice with OLE for 4 days had no effect on BDNF mRNA and protein expression in their PFC compared to controls (Figure 4A); however, FEN, a more potent PPARα agonist, up-regulated BDNF in this brain area (Figure 4A). The inducing effect of FEN on BDNF expression in the PFC was PPARα-dependent, because the drug did not affect BDNF expression in the PFC of *Pparα*-null mice (Figure 4A).

As in the case of BDNF, subacute treatment of WT mice with OLE had no effect on *TrkB* mRNA expression in their PFCs when compared to controls (Figure 4B). Interestingly though, OLE markedly repressed NT3 mRNA expression in the PFC of WT mice potentially via PPRAα activation, because no similar effect was observed in *Pparα*-deficient mice (Figure 4C). FEN up-regulated both NT3 and NT4/5 in the PFC of WT mice, and this effect appears to be PPARα-dependent, because it was not observed in *Pparα*-null mice (Figure 4C,D).

### 3.5. Assessment of Subacute OLE Treatment in Neural Plasticity Indices in Hippocampus

**Figure 5:**
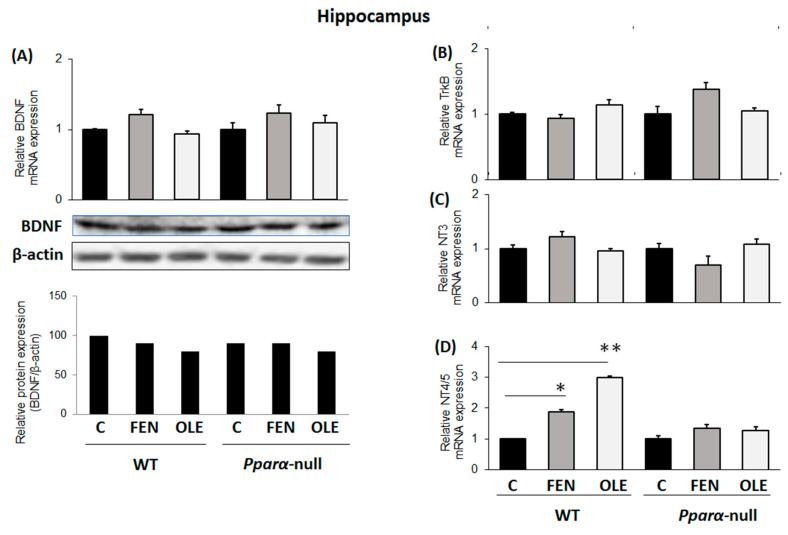
The effect of subacute treatment with oleuropein (OLE) on neural plasticity indices in the hippocampus of mice. (**A**) Following treatment with either OLE or fenofibrate (FEN), brain-derived neurotrophic factor (BDNF) mRNA levels were analyzed in 129/Sv wild-type (WT) and *Pparα*-null mice via qPCR, and protein levels using Western blot. (**B**) TrkB receptor mRNA and protein levels were also analyzed using qPCR and Western blot analysis, respectively. (**C**) Neurotrophin NT3 and (**D**) NT4/5 mRNA levels were also analyzed with qPCR. Values were normalized to β-actin, and are expressed as mean ± SE (n = 8–10). Comparisons were between controls C and OLE-treated mice. Treatment group differences were calculated using one-way ANOVA, followed by Bonferroni’s test. * *p* < 0.05, ** *p* < 0.01.

Treatment of mice with either OLE or FEN for 4 days had no effect on *BDNF*, *TrkB* and *NT3* expression in their hippocampus compared to controls (Figure 5A–C, respectively). Nonetheless, both drugs up-regulated NT4/5 in the hippocampus, and this up-regulation appears to be PPARα-mediated, because no NT4/5 up-regulation was induced by FEN and OLE in *Pparα*-null mice (Figure 5D).

### 3.6. Effect of OLE on Differentiated Human SH-SY5Y Neuroblastoma Cells

**Figure 6:**
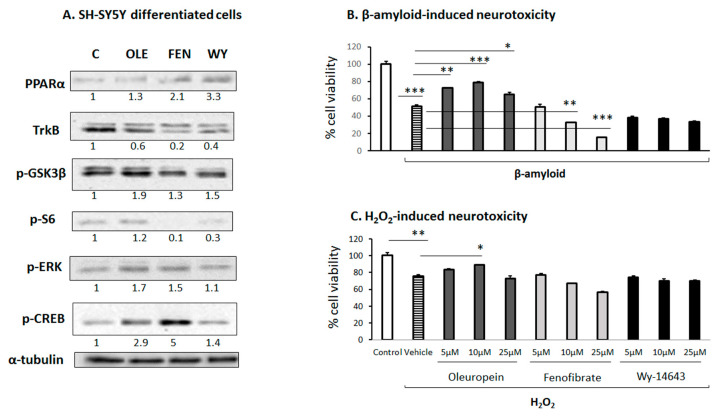
The effect of oleuropein (OLE), fenofibrate (FEN) and Wy-14643 (WY), PPARα agonists, on (**A**) PPARα and TrkB protein expression and on the phosphorylated expression levels of GSK3β, S6, ERK1/2 and PKA/CREB in differentiated human SH-SY5Y neuroblastoma cells into cholinergic neurons; (**B**) β-amyloid neurotoxicity and (**C**) H_2_O_2_-induced neurotoxicity. Phosphorylated GSK3β, S6, ERK1/2 and CREB expression levels were analyzed in proteins extracted from differentiated human SH-SY5Y cells using Western blot analysis. The numbers underneath the lanes represent the relative protein expressions that are defined as the ratio between the drug-treated and control expression, which is set at 1. Beta-amyloid- and H_2_O_2_-induced neurotoxicity was assessed using a spectrophotometric analysis of the samples at 492 nm to determine the percentage of cell viability. C: control (DMSO-treated cells). * *p* < 0.05, ** *p* < 0.01, *** *p* < 0.001.

An in vitro investigation using human SH-SY5Y neuroblastoma cells differentiated into cholinergic neurons indicated that OLE and FEN activated both the ERK1/2 and PKA/CREB signaling pathways (Figure 6A), whereas Wy-14643 did not affect these signaling pathways (Figure 6A). Moreover, all three substances induced *PPARα* expression in these cells. Interestingly, OLE increased the phosphorylation of GSK3β, whereas FEN and Wy-14643 had a weaker effect on it (Figure 6A).

It is noteworthy that preincubation of the differentiated SH-SY5Y cells with OLE (but not with FEN or Wy-14643) protected them most prominently from natural amyloid β (Aβ) peptides (Figure 6B), and to some extent from the H_2_O_2_-induced cell toxicity (Figure 6C). In particular, OLE at a concentration of 5–10 μM provided 50% neuroprotection against Aβ-induced toxicity. These OLE-induced neuroprotective effects appear to be PPARα-independent, because two other more selective PPARα agonists, Wy-14643 and FEN, either did not protect or even dose-dependently exaggerated the toxic effects of Aβ amyloid peptides, respectively (Figure 6B).

## 4. Discussion

The current study investigated the impact of OLE on neural plasticity in the hippocampus and PFC of mice, emphasizing the role of the nuclear receptor and transcription factor, PPARα. The findings indicated that chronic treatment of WT mice with OLE increased the synthesis of BDNF, as previously reported [59], and its receptor, TrkB, in their PFC as compared to controls; however, the drug had no effect on them in the PFC of *Pparα*-null mice. This finding underscores the crucial role of PPARα in the OLE-induced up-regulation of these important indices of neural plasticity in the PFC, which is potentially triggered by activation of the ERK1/2, AKT and PKA/CREB signaling pathways that possess crucial roles in the regulation of neurotrophins [4], neural plasticity [60,61] and survival [61,62,63,64]. The present findings are in line with those of a previous study reporting that plant compounds, including resveratrol and OLE among others, improve synaptic plasticity by activating neuronal signaling pathways, which control the memory and long-term potentiation (LTP) of synapses. The OLE-induced LTP in the hippocampus indicates increased synaptic activity, which is usually followed by a long-lasting increase in signal transmission among neurons, and is triggered by activation of signaling pathways including the PKA/CREB [29,65,66].

Unlike long-term OLE treatment, the subacute administration of WT mice with the drug at the given dose did not manage to increase BDNF and TrkB synthesis in their PFCs. Nonetheless, subacute treatment of WT mice with FEN, a selective PPARα agonist [67], up-regulated BDNF in their PFCs, but it did not affect *TrkB* expression in this brain tissue. The FEN-induced BDNF up-regulation in the PFC is PPARα-dependent, because the drug did not increase this neurotrophic factor in the PFC of *Pparα*-null mice. Interestingly, FEN also increased the synthesis of NT3 in the PFC of WT mice, and NT4/5 in both the PFC and hippocampus via PPARα activation, as no similar up-regulating effects were detected in *Pparα*-deficient mice. Both the long-term and subacute treatments with OLE had no up-regulating effect on *NT3* expression in the PFC and hippocampus of WT mice. Although subacute OLE administration had a down-regulating effect on *NT3* in the PFC of WT mice, the drug up-regulated NT4/5 in their hippocampus via a mechanism potentially involving PPARα activation, because it did not affect *NT4/5* expression in *Pparα*-null mice. Unlike previous studies indicating that OLE can improve synaptic plasticity in the dentate gyrus of the rat hippocampus, thus attenuating Alzheimer’s disease-like pathology [68], the present study indicated that either chronic or subacute treatment of WT mice with OLE at the given dose did not up-regulate the effect on BDNF and TrkB in their hippocampus; however, subacute OLE increased the synthesis of NT4/5 in this brain region. Apparently, the effect of OLE on neuronal plasticity indices is species-, dose- and time-dependent.

It is also noteworthy that OLE protected differentiated human SH-SY5Y neuroblastoma cells from β-amyloid- and H_2_O_2_-induced toxicity. This neuroprotective effect of OLE against β-amyloid-induced toxicity was unrelated to PPARα activation, because both selective PPARα agonists, FEN and Wy-14643, did not prevent neurotoxicity in this in vitro neuronal model. It appears that OLE may exploit both PPARα-dependent and independent pathways to promote neural plasticity and protect against oxidative stress and β-amyloid neurotoxicity. This hypothesis is supported by the findings of a previous study reporting that the neuroprotective effect of PPARα agonists do not necessarily directly depend on PPARα-regulated pathways [69]. The present findings, along with those from previous studies reporting that OLE prevents the aggregation of β-amyloids, tau, amylin, α-synuclein and ubiquitin proteins in the brain, reducing neuronal apoptosis and activating several antioxidant pathways [70], indicate that OLE and similar drugs such as hydroxytyrosol provide neuroprotection and could be used to prevent or delay the onset of neurodegenerative disorders, a subject that should be thoroughly investigated in the framework of clinical studies.

## 5. Conclusions

The present findings indicate that neuroprotection against oxidative stress and β-amyloid toxicity, as well as the induction of neural plasticity in several brain sites, belongs to the broad spectrum of the beneficial effects of OLE, the main constituent of olive products, a basic constituent of the Mediterranean diet. In this concept, OLE and similar drugs acting predominantly as PPARα agonists could modulate a diverse repertoire of functions in the central and peripheral nervous systems, as well as in non-neuronal tissues. Therefore, it is of particular interest to further investigate the potential beneficial effects of PPARα agonists on synaptic plasticity/function and dendritic outgrowth, which are critical parameters, among others, in the regulation of cognitive functions.

## Figures and Tables

**Table 1:** Oligonucleotide sequences for quantitation of gene mRNA concentration using quantitative PCR assays.

Gene	Sequences of Primers	
*BDNF*	F	5′-TGAGTCTCCAGGACAGCAAA-3′
	R	5′-GACGTTTACTTCTTTCATGGGC-3′
*TrkB*	F	5′-TGATGTTGCTCCTGCTCAAG-3′
	R	5′-CCCAGCCTTTGTCTTTCCTT-3′
*NT3*	F	5′-CGGATGCCATGGTTACTTCT-3′
	R	5′-AGTCTTCCGGCAAACTCCTT-3′
*NT4/5*	F	5′-AGCCGGGGAGCAGAGAAG-3′
	R	5′-CACCTCCTCACTCTGGGACT-3′

## Data Availability

All raw data of this study are available upon demand.

## Abbreviations

PPARα	peroxisome proliferator-activated receptor α
BDNF	Brain-derived neurotrophic factor
TrkB	tyrosine kinase receptor B
NT3	neurotrophic factor 3
NT4/5	neurotrophic factor 4/5
PGC-1α	peroxisome proliferator-activated receptor gamma coactivator-1 alpha
OLE	Oleuropein
FEN	Fenofibrate
WY	Wy-14643
PFC	prefrontal cortex
AD	Alzheimer’s disease
WT	wild type
